# Causal relationship between ADHD and frozen shoulder: Two-sample Mendelian randomization

**DOI:** 10.1097/MD.0000000000035883

**Published:** 2023-11-03

**Authors:** Guang-Hua Deng

**Affiliations:** a Ya’an Hospital of Traditional Chinese Medicine, Ya'an, Sichuan, China.

**Keywords:** ADHD, frozen shoulder, Mendelian randomization

## Abstract

To investigate the causal relationship between attention deficit and hyperactivity disorder (ADHD) and frozen shoulder using Mendelian randomization (MR). Data were pooled from large-scale genome wide association studies, and genetic loci that were independent of each other and associated with ADHD and frozen shoulder in people of European ancestry were selected as instrumental variables. Three MR analyses, inverse variance weighting, weighted median and MR-Egger, were used to investigate the causal relationship between ADHD and frozen shoulder. Heterogeneity and multiplicity tests were used, and sensitivity analyses were performed using the “leave-one-out” method to explore the robustness of the results. The inverse variance weighting results showed an OR (95 % CI) of 1.12 (1.00–1.25), *P* = .046, indicating a causal relationship between ADHD and frozen shoulder. And no heterogeneity and multiplicity were found by the test and sensitivity analysis also showed robust results. The present study used a two-sample MR analysis, and by analyzing and exploring the genetic data, the study showed that ADHD is a risk factor for developing frozen shoulder, and patients with ADHD are more likely to suffer from frozen shoulder.

## 1. Introduction

Frozen shoulder, also known as adhesive capsulitis,^[1]^ is a pathological condition characterized by pain and limitation of joint motion in the shoulder.^[2]^ There are usually no significant findings in the patient’s history, clinical examination, or imaging evaluation to explain the loss of motion or pain.^[3,4]^ In recent years, many studies have found that attention deficit and hyperactivity disorder (ADHD) is associated with the development of a variety of disorders, such as synovitis, tenosynovitis, polyarthritis, and neck or shoulder pain.^[5]^ However, studies on ADHD and frozen shoulder are more lacking, and the causal relationship is uncertain. The causal relationship between ADHD and frozen shoulder still needs further investigation.

Mendelian randomization (MR), a genetic epidemiological method, is a useful tool to assess the causal role of ADHD and frozen shoulder.^[6]^ By using genetic variants such as single nucleotide polymorphisms (SNPs) as instrumental variants that can modify disease risk factors or exposures, MR studies can enhance causal inference of exposure-outcome associations.^[7]^ According to Mendel law of inheritance, genetic variants are not susceptible to confounding factors as they are randomly assigned during gamete formation.^[8]^ In addition, confounders and reverse causation can be minimized as genotypes cannot change as the disease progresses.^[9]^

To this end, we conducted a two-sample MR study to examine the association of ADHD with causality in frozen shoulder. We aimed to provide significant evidence for the causal role of ADHD in causing frozen shoulder.

## 2. Information and methods

### 2.1. Data sources

Genome-wide association study (GWAS) data for ADHD and frozen shoulder were obtained from the IEU OpenGWAS project (mr cieu.ac.uk) website. The website was accessed on 06 June, 2023.All data ultimately obtained for this study were from European populations. This includes ADHD (ieu-a-1183) with 8,047,420 SNPs, 35,191 in the observation group and 55,374 in the control group, and frozen shoulder (ebi-a-GCST90000512) with 15,184,371 SNPs and a sample size of 451,099. This study was a reanalysis of previously collected and published publicly available data and therefore did not require additional ethical approval.

### 2.2. Condition setting for SNPs as instrumental variables

The instrumental variable was highly correlated with exposure, with *F* > 10 as a strong correlation criterion.^[10]^ The instrumental variable is not directly related to the outcome but only affects the outcome through exposure; that is, there is no genetic pleiotropy. In this study, the intercept term of the MR-Egger regression model was nonzero (*P* < .05), indicating the absence of genetic pleiotropy.^[11]^ Instrumental variables were not associated with untested confounding.^[12]^ The human genotype–phenotype association database Phenoscanner V2 was searched for phenotypes associated with the instrumental variables at the genome-wide significance level to determine whether these SNPs were associated with potential risk factors.^[13]^

### 2.3. SNP screening

Significant SNPs were screened from the GWAS pooled data of ADHD (*P* < 5 × 10^−8^ was used as the screening condition)^[14]^; the linkage disequilibrium coefficient r^2^ was set to be 0.001 and the width of the linkage disequilibrium region to be 10,000 kb to ensure that the individual SNPs were independent of each other.^[15]^ The above screened ADHD-related SNPs were extracted from the GWAS pooled data of frozen shoulder, while SNPs directly related to outcome indicators were excluded (*P* < 5 × 10^−8^). The *F*-value of each SNP was calculated, and SNPs with weak instrumental variables (*F*-value < 10) were excluded.^[16]^ And the human genotype–phenotype association database was queried to screen for potentially relevant risk factor SNPs and exclude them.^[17]^

### 2.4. Methods of causality verification

The causal relationship between exposure (ADHD) and outcome (frozen shoulder) was verified using mainly inverse variance weighted (IVW) as, supplemented by MR-Egger and weighted median MR analyses with SNP as instrumental variables.

### 2.5. Sensitivity analysis

Sensitivity analyses were performed using several methods. First, the Cochran Q test was used to assess the heterogeneity between the individual SNP estimates, and a statistically significant Cochran Q test proved that the analyses were significantly heterogeneous. Second, Mendelian randomization pleiotropy residual sum and outlier (MR PRESSO) was used to validate the results in the IVW model, to correct for the effect of outliers, and if outliers existed, they were removed and the analysis was repeated. Third, the horizontal multiplicity of SNPs was tested using the MR Egger intercept test (MR Egger intercept test), and if the intercept term in the MR Egger intercept test analysis was statistically significant, it indicated that the MR analysis had significant horizontal multiplicity. Fourth, “leave-one-out” sensitivity analyses were performed by removing a single SNP at a time to assess whether the variant drove the association between the exposure and outcome variables. Fifth, funnel plots and forest plots were constructed to visualize the results of the sensitivity analyses. *P* < .05 suggests that there is a potential causal relationship in the MR analyses, which is statistically significant. All statistical analyses were performed using the “TwoSampleMR” package in R software version 4.3.0.

## 3. Results

### 3.1. Instrumental variables

In this study, 12 SNPs that were strongly associated with ADHD (*P* < 5 × 10^−8^) without chain disequilibrium (r^2^ < 0.001, kb = 10,000) were screened out. 12 SNPs were left by taking the intersection with SNPs in the pooled GWAS data for periarthritis, and by excluding SNPs that were directly associated with the outcome metrics. in our study, the *F*-value of each SNP was >10, indicating the absence of weak instrumental variables (see Table 1 for details). We searched the human genotype–phenotype association database and found no potentially relevant risk factor SNPs.

**Table 1:** Information on the final screening of ADHD SNPs from GWAS data (n = 12).

ID	SNP	Effect_Allele	Other_Allele	β	SE	*P*	*F*
1	rs10262192	A	G	0.073204	0.0132	2.89E−08	30
2	rs112984125	A	G	−0.106005	0.0146	3.58E−13	52
3	rs11591402	A	T	−0.0929051	0.0164	1.34E−08	32
4	rs1222063	A	G	0.0962007	0.0174	3.07E−08	30
5	rs1427829	G	A	−0.0799012	0.0133	1.82E−09	36
6	rs212178	A	G	−0.1154	0.02	7.68E−09	33
7	rs281324	C	T	0.0744973	0.0134	2.68E−08	30
8	rs28411770	C	T	−0.0861043	0.0151	1.15E−08	32
9	rs4858241	G	T	−0.0789036	0.014	1.74E−08	31
10	rs4916723	C	A	0.0766003	0.0135	1.58E−08	32
11	rs74760947	G	A	0.179797	0.0317	1.35E−08	32
12	rs9677504	A	G	0.116903	0.0206	1.39E−08	32

### 3.2. Causal relationship between ADHD and frozen shoulder

**Figure 1.**
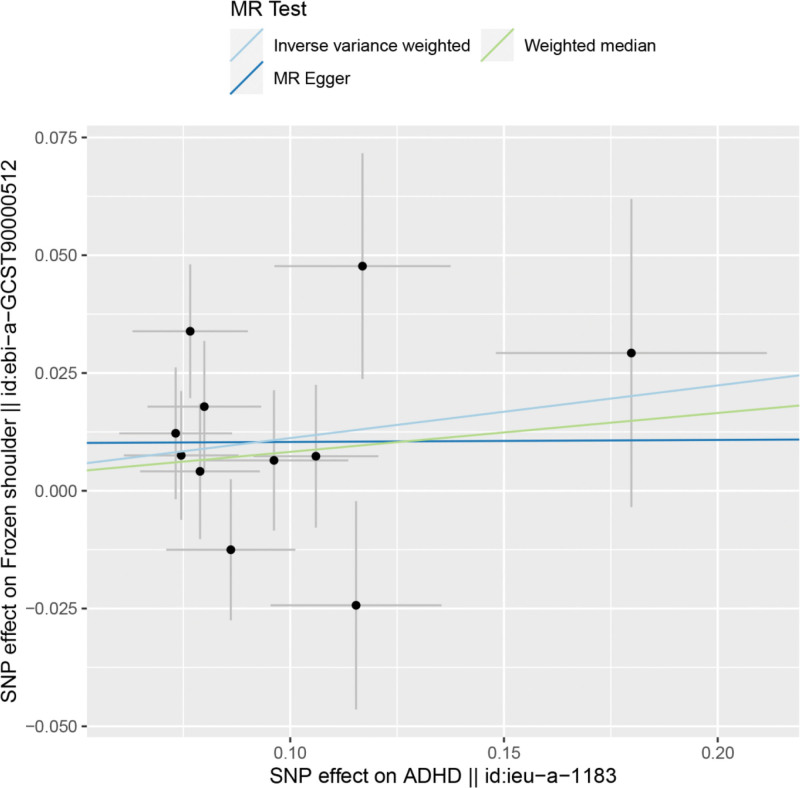
Scatter plot of ADHD and frozen shoulder. ADHD = attention deficit and hyperactivity disorder.

**Figure 2.**
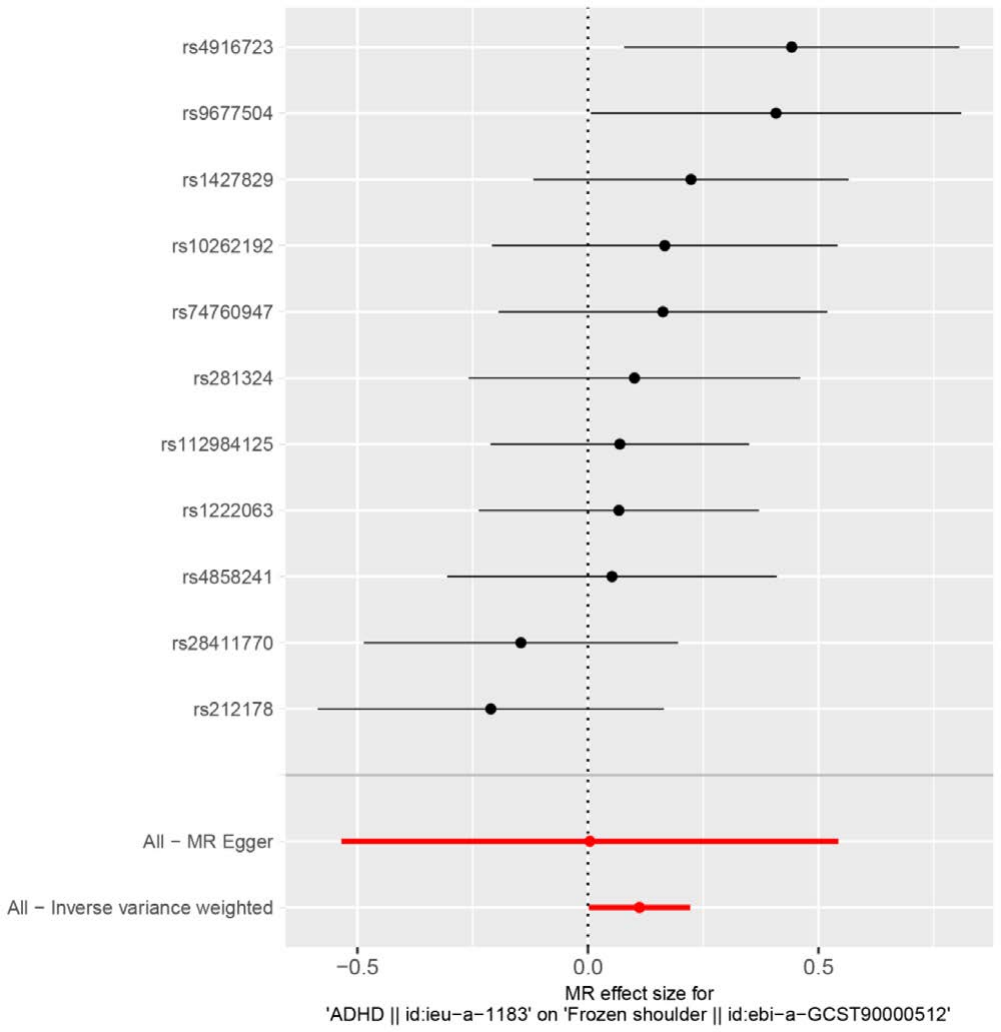
Forest plot of ADHD and frozen shoulder. ADHD = attention deficit and hyperactivity disorder.

The results of IVW by MR analysis showed that there was a causal relationship between ADHD and frozen shoulder. IVW:OR = 1.12, 95% CI = 1.00–1.25, *P* = .046 (see Table 2 for details). We can see from both the scatter plot (Fig. 1) and the forest plot (Fig. 2) that ADHD increases the risk of developing frozen shoulder.

**Table 2:** MR regression results of the 3 methods.

Method	β	SE	OR (95%CI)	*P*
IVW	0.112	0.056	1.12 (1.00–1.25)	.046
WME	0.083	0.073	1.09 (0.94–1.25)	.259
MR-Egger	0.004	0.275	1.00 (0.59–1.72)	.988

### 3.3. Sensitivity analysis

**Figure 3.**
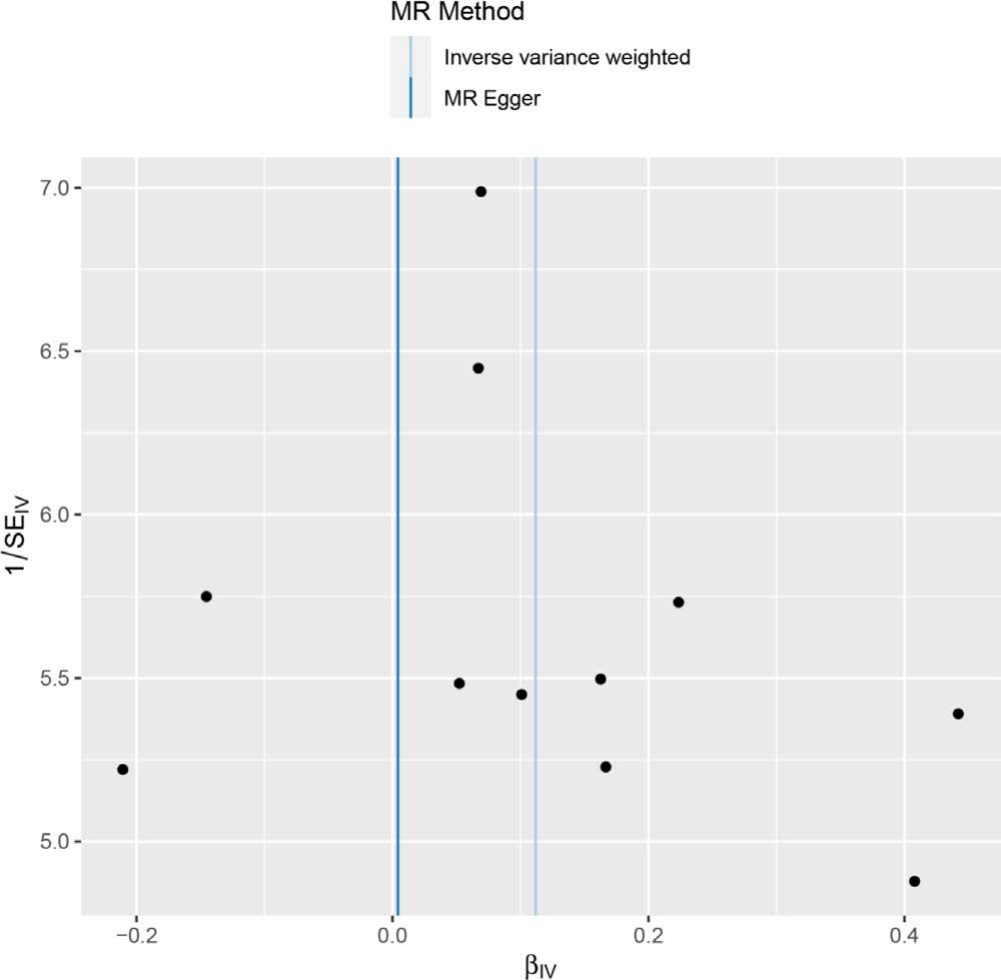
Funnel plot of ADHD and frozen shoulder. ADHD = attention deficit and hyperactivity disorder.

**Figure 4.**
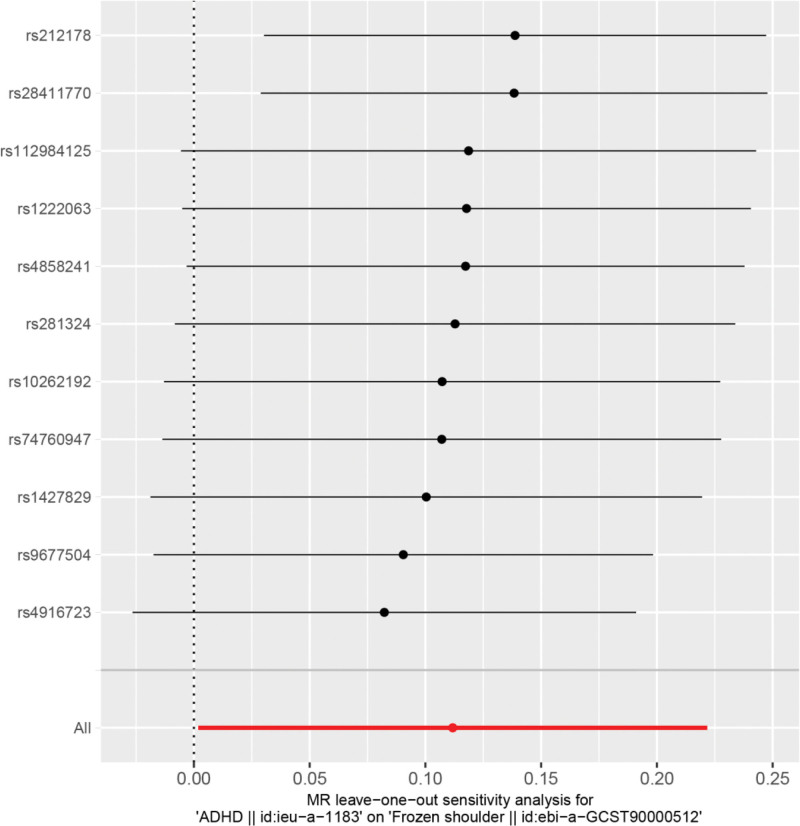
Analysis of ADHD and frozen shoulder by the leave-one-out method. ADHD = attention deficit and hyperactivity disorder.

Heterogeneity was tested using the IVW method (Cochran Q test, *P* = .348), and the results suggested that there was no heterogeneity. A funnel plot was drawn to show the heterogeneity results, as shown in Figure 3. MR-PRESSO was used to screen for SNPs that could lead to heterogeneity, and the results did not reveal any SNPs that would lead to heterogeneity in the results. The result of Global test by MR-PRESSO suggested that there was no pleiotropy (*P* = .698). The “leave-one-out” method uses the IVW method by default, and as can be seen in Figure 4, no single SNP will have a large impact on the overall results after eliminating any SNP, indicating that the results are robust.

## 4. Discussion

It is known that ADHD may be an observational risk factor for frozen shoulder, but the causality of this association is unclear. Our MR study aimed to reveal the causal relationship between ADHD and frozen shoulder. The two-sample MR results showed a causal association between ADHD and frozen shoulder, with an OR (95 % CI) of OR = 1.12, 95 % CI = 1.00–1.25, *P* = .046, indicating that people with AHDH have 1.12 times the risk of developing frozen shoulder compared to the general population.

The term “frozen shoulder” is often incorrectly used^[18,19]^ and attributed to other shoulder limitations such as rotator cuff tears or osteoarthritis. Subacromial lesions (e.g., rotator cuff tendinopathy, subacromial bursitis, and impingement syndrome) may also closely resemble frozen shoulder in the early stages.^[20]^ For appropriate management, it is important for the physician to establish the diagnosis. Frozen shoulder is estimated to affect 2% to 5% of the general population,^[21]^ and can be significantly painful and disabling. It most often affects people between the ages of 40 and 60 and occurs more often in women than in men.^[22,23]^ Therefore, it is necessary and urgent to identify risk factors and develop effective public strategies to prevent frozen shoulder. Adverse lifestyle behaviors are clear risk factors for frozen shoulder.^[24]^ Previous studies have reported that ADHD is a risk factor for many diseases such as iron deficiency anemia, obesity, type 2 diabetes mellitus, synovitis, tenosynovitis, polyarthritis, neck and shoulder pain.^[5]^ Similarly, the present study confirms that ADHD is positively associated with the risk of frozen shoulder from a genetic point of view. In addition statistical evidence from sensitivity analyses strongly supports our findings. Therefore, patients with ADHD should be treated promptly. Furthermore, screening for frozen shoulder should be increased in the ADHD population, so that patients with frozen shoulder can be detected early and treated in a timely manner, which is conducive to the prognosis of the patients.

The following suggestions can be made to address the fact that people with ADHD are more likely to suffer from frozen shoulder. Firstly, it is important for people with ADHD and their families to provide education and awareness support about frozen shoulder. They need to be aware of the symptoms, preventive measures and treatments of frozen shoulder so that they can take timely action. Secondly, people with ADHD are often characterized by hyperactivity and they need moderate physical activity to release energy and anxiety. However, excessive exercise and incorrect posture may increase the risk of frozen shoulder. Therefore, there is a need to encourage people with ADHD to choose sports that are suitable for them, and to instruct them on proper exercise posture and ways to protect their shoulders. Finally, scientific research on the relationship between ADHD and frozen shoulder should be strengthened to explore in depth the mechanisms and influencing factors. At the same time, public awareness of ADHD and frozen shoulder should be raised through publicity and education programmes to promote early prevention and intervention.

At the same time, there are some limitations in this study. Firstly, since all the data came from a population of European origin, the results do not represent a truly randomized population sample and are not applicable to other races. Secondly, although various sensitivity analyses have been performed in this study to test the hypotheses of the MR study, it is difficult to completely rule out horizontal pleiotropy of instrumental variables. Finally, the current sample size of GWAS data is still not large enough, and more in-depth studies using more GWAS data are needed in the future.

## 5. Conclusion

In conclusion, this study used a two-sample MR analysis, and by analyzing and exploring the genetic data, the study showed that ADHD is a risk factor for developing frozen shoulder.

## Author contributions

**Conceptualization:** Guang-Hua Deng.

**Data curation:** Guang-Hua Deng

**Formal analysis:** Guang-Hua Deng.

**Funding acquisition:** Guang-Hua Deng.

**Investigation:** Guang-Hua Deng.

**Methodology:** Guang-Hua Deng.

**Project administration:** Guang-Hua Deng.

**Resources:** Guang-Hua Deng.

**Software:** Guang-Hua Deng.

**Supervision:** Guang-Hua Deng.

**Validation:** Guang-Hua Deng.

**Visualization:** Guang-Hua Deng.

**Writing – original draft:** Guang-Hua Deng.

**Writing – review & editing:** Guang-Hua Deng.
